# Degradation properties of a biodegradable shape memory elastomer, poly(glycerol dodecanoate), for soft tissue repair

**DOI:** 10.1371/journal.pone.0229112

**Published:** 2020-02-21

**Authors:** Harsha Ramaraju, Loran D. Solorio, Martin L. Bocks, Scott J. Hollister

**Affiliations:** 1 Wallace H. Coulter Department of Biomedical Engineering, Georgia Institute of Technology, Atlanta, Georgia, United States of America; 2 Department of Biomedical Engineering, University of Michigan, Ann Arbor, Michigan, United States of America; 3 UH Rainbow Babies & Children’s Hospital, School of Medicine, Case Western Reserve University, Cleveland, Ohio, United States of America; LAAS-CNRS, FRANCE

## Abstract

Development of biodegradable shape memory elastomers (SMEs) is driven by the growing need for materials to address soft tissue pathology using a minimally invasive surgical approach. Composition, chain length and crosslinking of biocompatible polymers like PCL and PLA have been investigated to control mechanical properties, shape recovery and degradation rates. Depending on the primary mechanism of degradation, many of these polymers become considerably stiffer or softer resulting in mechanical properties that are inappropriate to support the regeneration of surrounding soft tissues. Additionally, concerns regarding degradation byproducts or residual organic solvents during synthesis accelerated interest in development of materials from bioavailable monomers. We previously developed a biodegradable SME, poly(glycerol dodecanoate) (PGD), using biologically relevant metabolites and controlled synthesis conditions to tune mechanical properties for soft tissue repair. In this study, we investigate the influence of crosslinking density on the mechanical and thermal properties of PGD during *in vitro* and *in vivo* degradation. Results suggest polymer degradation *in vivo* is predominantly driven by surface erosion, with no significant effects of initial crosslinking density on degradation time under the conditions investigated. Importantly, mechanical integrity is maintained during degradation. Additionally, shifts in melt transitions on thermograms indicate a potential shift in shape memory transition temperatures as the polymers degrade. These findings support the use of PGD for soft tissue repair and warrant further investigation towards tuning the molecular and macromolecular properties of the polymer to tailor degradation rates for specific clinical applications.

## Introduction

Regenerative therapies involving minimally invasive surgical procedures require materials to mechanically bridge tissue defects while allowing implant delivery through smaller incisions or via transcatheter approach. Composites of conventional thermoplastic polymers and hydrogels along with manufacturing innovation can tailor initial material stiffness to match surrounding tissues but often demonstrate unsatisfactory clinical outcomes[1–4]. When injected *in vivo*, hydrogels have been prone to migration and biodegradable polymers like PLGA, PLA and PCL predominantly undergo bulk degradation causing rapid changes in mechanical integrity during the degradation timeframe [5]. Biodegradable elastomers like polyglycerol sebacate(PGS) and poly-diol citrates have been increasingly studied for soft tissue repair applications [6–9]. These materials are synthesized from components of common metabolic pathways and consequently break down into biologically compatible byproducts. The shape transition temperatures (7–42°C), elasticity (300–500% strain at break) and tensile modulus (0.1–80 MPa) can be tuned to make these materials ideal for a wide range of soft tissue repair including but not limited to neural, cardiac and musculoskeletal pathologies [10].

Additionally a new class of polymers, biodegradable shape memory elastomers (SMEs), are able to address the clinical requirements for improved handling and ease of use for minimally invasive surgeries[11–14]. These polymers can be compressed into a smaller temporary shape to traverse keyhole incisions or intravascular delivery sheaths, but can expand upon being delivered into the body via a triggering cue to resume their elastomeric properties and the initial shape.

The difference in ambient and body temperature is the primary triggering cue of thermally responsive SMEs for biomedical use. Block-copolymerization, chain softening diols or isothiocyantes, and composite polymer networks have been utilized to improve the elasticity and reduce the degradation time of the polymers while providing functional domains that mediate chemical crosslinks driving the overall shape memory behavior[4,15,16]. Chain length and polymer blending have also been used to drive physical crosslinks to encourage shape memory behavior while reducing the longer degradation times compared to base thermoplastic polymers or chemically crosslinked polymers[17–19]. For instance, composite PCL-PLLA oligo diol based shape memory elastomers, PEG-PCL diblock co-polymers, PCL linear and brush architectures using diacrylated or norbornene PCL backbones, and PCL-DA/PLLA interpenetrating networks were used to tailor macromolecular structures, mechanical properties, and degradation times. These polymers utilize faster degrading co-polymers, physical crosslinks, interpenetrating networks, and macromolecular architecture to control the overall mechanical properties and degradation rates. However, degradation behavior of physical crosslinks is highly variable depending on the composition and the byproducts have been reported to cause mild to moderate inflammation over a one year timeframe[20]. Additionally, synthesis and manufacture of these shape memory elastomers often requires organic solvents.

We previously manufactured a thermally triggered biodegradable SME from a polycondensation of glycerol and dodecanedioic acid(PGD) with thermally formed chemical crosslinks[21]. Similar to PGS and citrate-based polymers, synthesis and formation of chemical crosslinks involves bioavailable metabolites to address biocompatibility and cytotoxicity concerns. At 37°C, PGD exhibits nonlinear elastic mechanical properties with tangent moduli between 0.5 to 5 MPa exhibiting 70–80% elastic deformation appropriate for various cardiovascular and orthopedic soft tissue repair applications. By changing the crosslink density, we can further tune the mechanical and shape recovery properties[22]. The primary aims of this study were to investigate the effect of crosslink density on the degradation rate and the ensuing mechanical and thermal properties post-degradation *in vitro* and in a subcutaneous mouse model.

## Materials and methods

University of Michigan Committee for the Use and Care of Animals approved this study. Animals were anesthetized by isoflurane and no analgesics were required during recovery surgery or followups. Animals were sacrificed by CO_2_ asphyxiation and cervical dislocation.

### PGD synthesis and implant preparation

**Fig 1:**
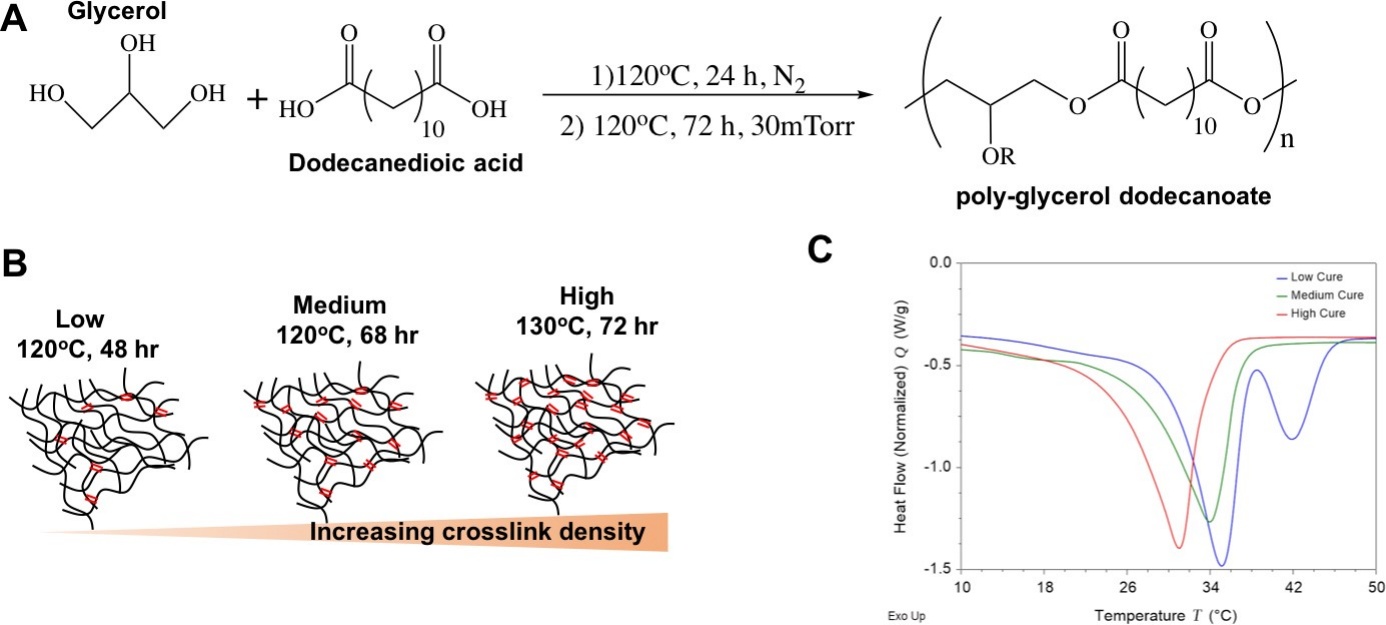
Synthesis and thermal characterization of PGD. **A)** Reaction scheme for PGD synthesis leading to the formation of a PGD pre-polymer with R being a hydrogen bond or a carbon bond with another PGD polymer chain **B)** Schematic of crosslink density **C)** DSC thermogram of low, medium and high cure PGD.

Polyglycerol dodecanoate was synthesized as previously described[21,22] (**Fig 1A**). The R groups form crosslinks. Briefly, equimolar amounts of glycerol (MP Biomedical, LLC, Solon OH) and dodecanedioic acid (Sigma-Aldrich, St. Louis MO) were mixed at 120°C under nitrogen for 24 h. The reaction was then switched to vacuum at 30 mTorr at 120°C for an additional 24 h. Pre-polymer was subsequently poured into rectangular silicone molds adjusting volume to produce 2mm thick specimens. Molds were cured in a vacuum oven for 48hr at 120°C for low cure (lPGD), 72hr at 120°C for medium cure (mPGD) and 48 hr at 130°C for high cure PGD (hPGD). Cured samples were subsequently removed from silicone molds and cut to appropriate size with an 8mm punch biopsy. Samples were ethanol sterilized, washed in PBS and dried prior to implantation.

### Differential scanning calorimetery

Differential Scanning Calorimetry (DSC) was conducted using a Discovery Q250 with an RCS90 cooling system (TA instruments, New Castle DE). Samples(n = 4) were dried in a vacuum desiccator, weighed, and placed in a Tzero® pan. Samples were preheated to 90°C from 25°C at a rate of 10°C/min to remove thermal history. After an isothermal hold for 3min, samples were cooled at a rate of 5°C/min to -50°C and heated back up to 70°C at a rate of 5°C/min. Thermal transitions were measured using the TA instruments analysis software.

### *In vitro* accelerated hydrolytic degradation

Degradation studies were conducted as previously described[23]. Briefly, samples (n = 5) were cut to 8mm diameter and 2mm thickness and weighed to determine initial mass. Hydrolytic degradation of hPGD, mPGD, and lPGD was conducted by placing each individual sample in 20mL of 0.1mM NaOH at 37°C for 2, 4, 8, or 18 weeks. Samples were washed in high purity water (HPW), dried at 40°C for 7 days and weighed to determine mass loss.

### *In vivo* degradation in a subcutaneous mouse model

All surgical procedures were performed in accordance with NIH guidelines for the care and use of laboratory animals (NIH Publication #85‐23 Rev. 1985) and the University of Michigan's Committee on Use and Care of Animals. Outbred C57Bl6 mice (C57bl/6, Jackson Labs) weighing between 15–20 g (n = 16) were anesthetized with isoflurane in O_2_ (5% induction and 2% maintenance at 1 mL min^−1^). A midline longitudinal incision was made on the back of each mouse and four pockets (two on each side) were made in the subcutaneous tissue beneath the dorsal skin. Scaffolds were randomly placed into each pocket and the incision was closed with surgical staples and no post-operative analgesics were required. Animals were sacrificed after 1 month and 4 months postoperatively by CO_2_ asphyxiation and cervical dislocation and transplants were harvested. Harvested implants (n = 8 per group) were cleaned of surrounding tissue, rinsed sequentially in PBS and ddH_2_O the surface adsorbed water was removed. Implants were subsequently weighed and the wet weights were recorded. Implants were then dried at 40°C for 7 days and the dry weight was measured. Swelling percentage was calculated as 100 x (*W*_w_ − *W*_d_)/*W*_d_ where *W*_w_ is the wet weight and *W*_d_ is the dry weight of the sample.

### Mechanical testing

Explants were cleared of surrounding tissue, washed in sterile saline, and dried at 40°C for 7 days as described above. Compression testing was conducted as reported previously[22]. Briefly, 6-mm PGD disks within a custom-built temperature control chamber were tested using an MTS system equipped with a 500 N load cell and a porous metal platen. Samples (N = 8) were tested at 37°C at a compression rate of 2 mm/min. Samples were compressed to 60% strain 37°C.

### Statistical analysis

Statistical analysis was conducted using JMP 13.1 (SAS Inc., Atlanta GA). Additionally, two-way ANOVA on ranks using Bonferonni post-hoc tests for all pairwise comparisons were done in Graphpad Prism (Graphpad Software Inc., La Jolla CA) to analyze *in vitro* and *in vivo* degradation rates.

## Results

Crosslink density of thermally cured PGD can be varied by altering temperature and duration of the curing process (**Fig 1B**) thereby providing control over thermal transitions and mechanical properties. The role of crosslink densities on the *in vitro* and *in vivo* degradation behavior of the polymer was investigated.

### High cure PGD has a lower T_trans_ compared to medium cure PGD and low cure PGD

Differential scanning calorimetry indicates a lower transition temperature for hPGD compared to lPGD and mPGD (**Fig 1C and Table 1**). The increase in crosslinking results in a decrease in transition temperatures. The low cure PGD exhibits two melt transitions(**Fig 1C**), indicating two distinct molecular architectures within the polymer matrix in large part driven by varying degrees of crystallinity and crosslink density. Additionally, the increase in crosslink density decreases the flexibility in the polymer network to arrange into crystalline lamellae. Consequently, the overall crystallinity of the polymer decreases observed by the relative decrease in the enthalpy of fusion **(Δ H**_**m**_**)** with a corresponding increase crosslink density (**Table 1)**

**Table 1:** PGD melt (T_m_) and recrystallization (T_c_) transition temperatures and enthalpy of fusion (Δ H_m_).

Cure Condition	T_m_(^o^C)	T_c_(^o^C)	Δ H_m_ (J/g)
Low	39.3 ± 0.2	26.3± 1.8	45.3±1.2
Medium	36.9 ± 0.8	26.6± 0.5	37.9±0.8
High	34.6 ± 0.5	27.9± 1.3	32.2±1.9

### High cure PGD degrades faster under accelerated degradation conditions *in vitro*

**Fig 2:**
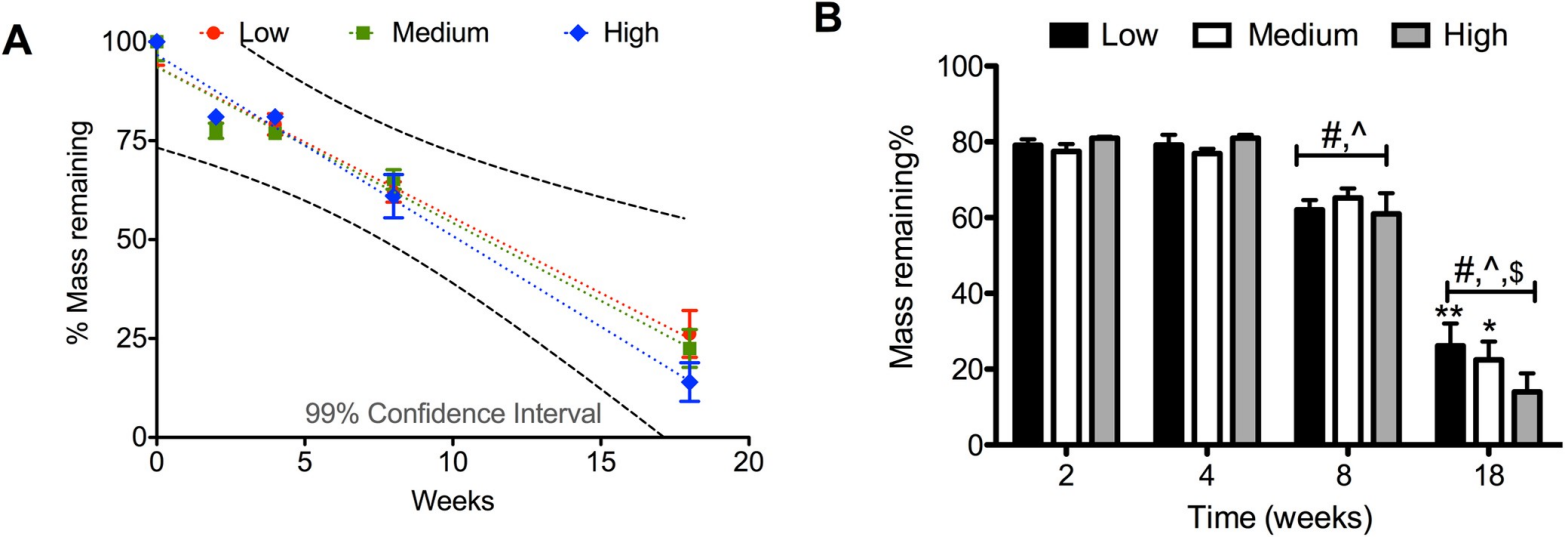
*In vitro* hydrolytic degradation of PGD over 8 weeks A)linear mass loss and possible complete degradation over the 18 week timeframe B) differences amongst groups and timepoints demonstrated greater mass loss at weeks 8 and 18 compared to week 2 and week 4 across all groups (p<0.001) and greater mass loss at week 18 compared to weeks 2, 4, and 8 across all groups (p<0.001) hPGD mass loss was significantly greater than both mPGD, * (p < 0.01), and lPGD ** (p < 0.001). # less than week 2 (p<0.001), ^ less than week 4 (^, p <0.001), **†** less than week 8 (p<0.001).

*In vitro* hydrolytic degradation in 0.1mM NaOH(**Fig 2A**) demonstrates a near linear degradation profile over an 18 week time frame. Linear regressions failed significance, but data bounded within the 95% CI bands suggest possibility of complete degradation within the tested timeframe (**Fig 2A**). Comparing differences across cure conditions and timepoints (**Fig 2B**) revealed that hPGD degraded faster than mPGD (p <0.01) and lPGD (p<0.001) by week 18. Mass loss for all curing conditions was significantly greater at week 8 and week 18 compared to week 2 (p<0.001) and week 4 (p <0.001). As expected mass loss was also greater overall by week 18 compared to week 8 (p<0.001).

### PGD *in vivo* degradation is dominated by surface erosion

**Fig 3:**
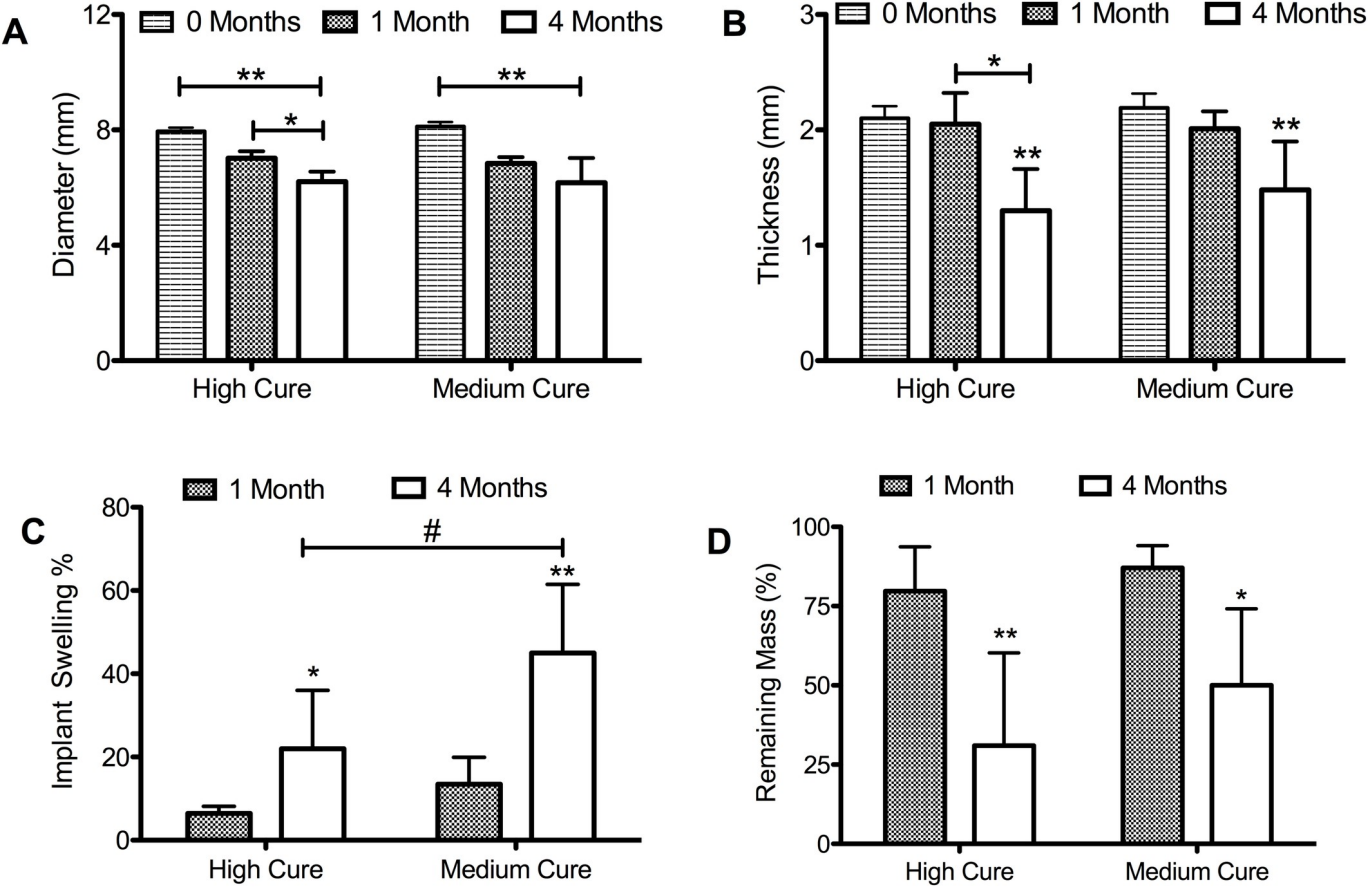
Degradation of mPGD and hPGD after 1 and 4 months of subcutaneous *in vivo* implantation. **a)** Total decrease in diameter compared to undegraded controls (** p < 0.001) compared to 1 month(*p<0.01) **b)** Total decrease in thickness compared to non-degraded controls (** p < 0.1) **c)** Mass loss percentage after 1 month and 4 month *in vivo* degradation (* p < 0.01, ** p<0.001) **d)** Total mass loss normalized to implant surface area (p <0.05).

Samples decreased proportionately in thickness and diameter, suggesting a mechanism of surface erosion (**Fig 3A and 3B**). Greater crosslinking in hPGD contributes to less swelling compared to mPGD (**Fig 3C**, p < 0.01, p<0.001). Swelling increases in mPGD and hPGD by month 4 of *in vivo* degradation compared to month 1 of *in vivo* degradation. This indicates a degradation of crosslinks during the timeframe of *in vivo* implantation. As expected, we see greater percentage mass loss of PGD after 4 months *in vivo* degradation compared to 1 month for both mPGD (p <0.01) and hPGD (p<0.001, **Fig 3D**). There were no significant differences in mass or volume loss between mPGD and hPGD.

### PGD thermal properties change non-linearly during degradation

**Fig 4:**
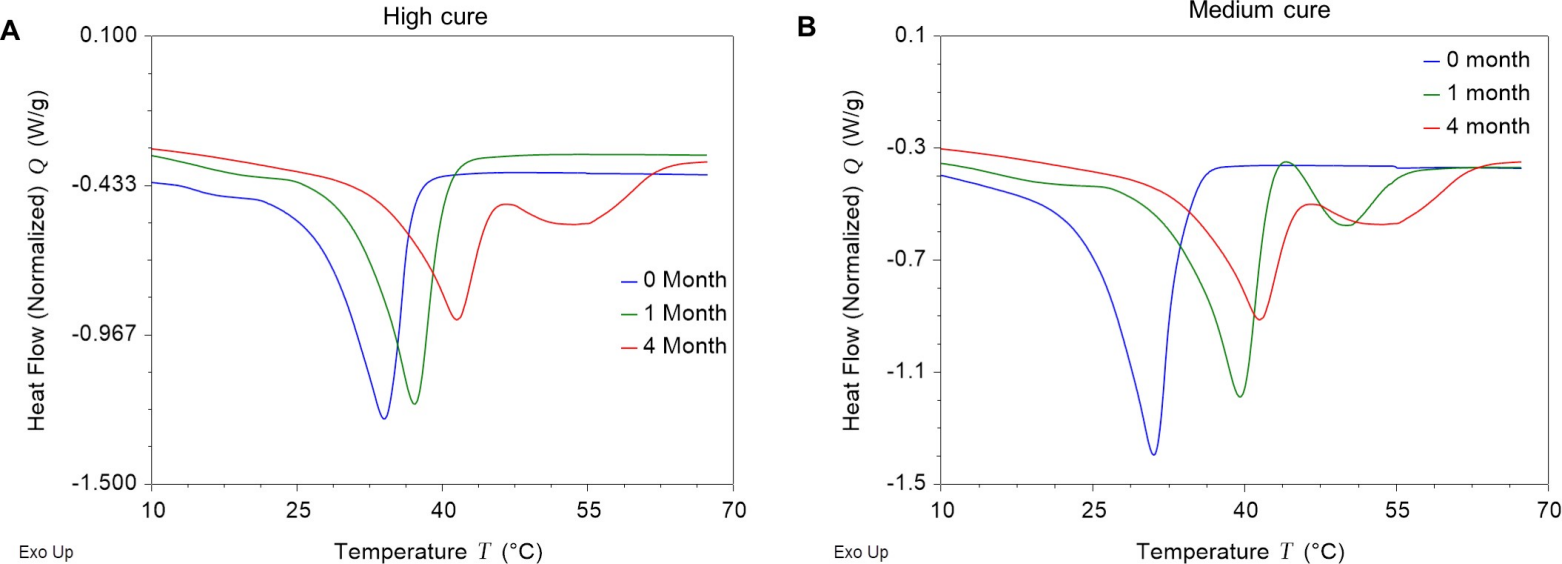
PGD thermal properties post-degradation **A)** Melt transition of high cure PGD **B)** Melt transition of medium cure PGD.

**Fig 5:**
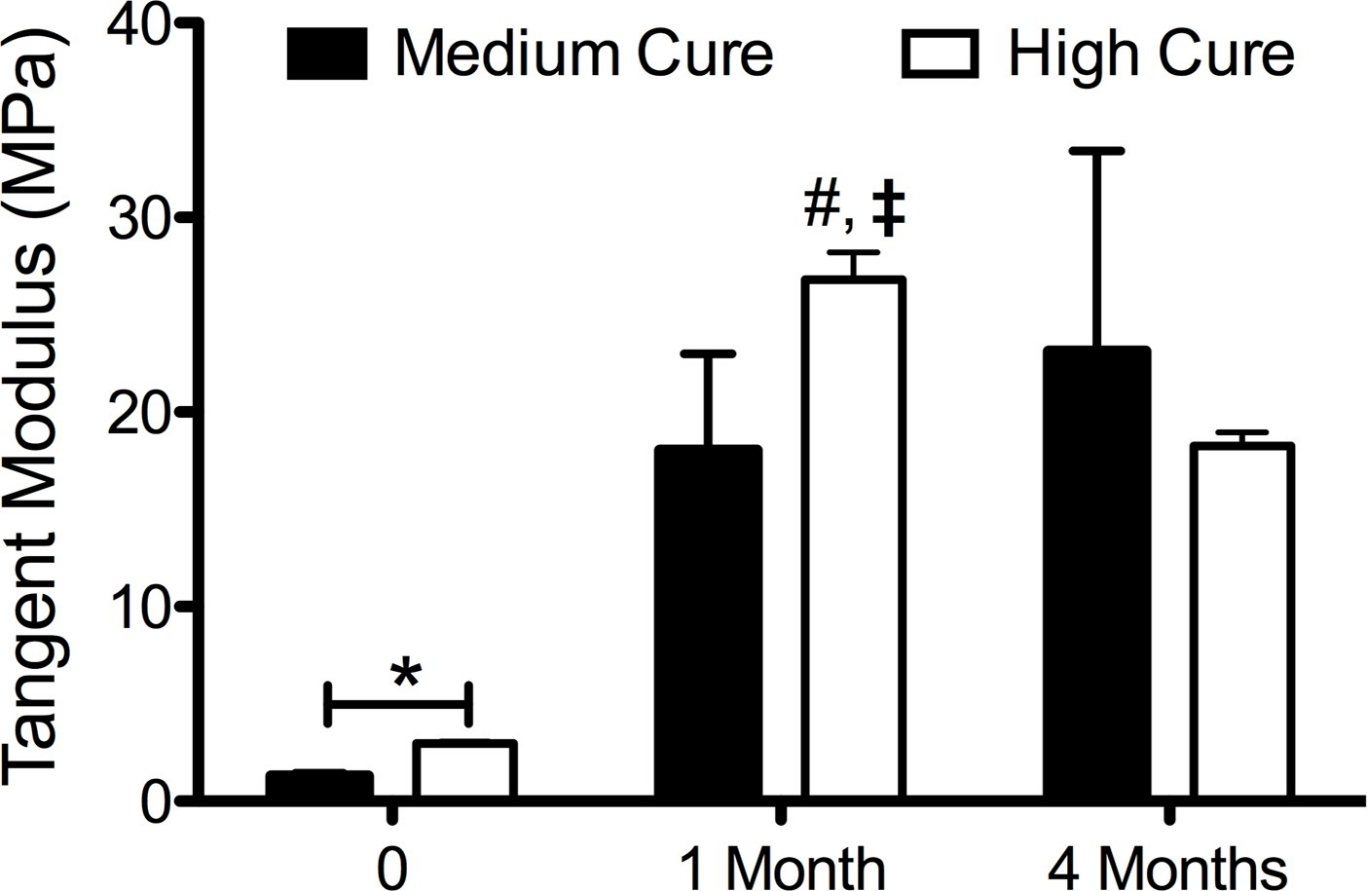
Mechanical properties of post-degraded PGD implants calculated by measuring the tangent modulus at 12.5% strain. Control implants are less stiff than implants from 1 month and 4 months (p< 0.001). High cure implants are stiffer than medium cure implants after 1 month (p <0.01). High cure implants at 1 month are stiffer than high cure implants after 4 months (**‡,** p <0.01).

Changes in intrinsic material properties during degradation are reflected in the DSC thermograms of the recovered PGD samples post-implantation at 1 month and 4 months (**Fig 4**). Increase in transition temperature between 1 month and 4 month indicates a degradation of crosslinks which coincides with water swelling (**Table 2**). The difference in DSC thermograms between control, 1 month and 4 months indicates a gradual decrease in crosslinking especially visible in the 4 month melt transition which is accompanied by decomposition peak of the polymer. With sufficient breakdown of the polymer crosslinks, the amorphous regions melt and decompose during the thermal cycle. This shift in melt transition temperatures accompanied by a decomposition curve is even more evident in the medium cure samples which have a lower initial number of crosslinks(**Fig 4B**). Moreover, Δ H_m_ decreases as the polymer degrades indicating decreased crystallinity in both high cure and medium cure PGD. This decrease in relative crystallinity occurs disproportionately between high cure and medium cure samples with mPGD losing more crystalline domains in the first month and hPGD losing more crystalline domains by 4 months (**Table 2**). The stiffness also changes disproportionately between medium cure and high cure PGD(**Fig 5**).

**Table 2:** PGD thermal properties after *in vivo* degradation.

Cure Condition	Timepoint(months)	T_m_(^o^C)	T_c_(^o^C)	Δ H_m_ (J/g)
High	0	37.1± 0.3	25.02± 0.4	32.2±1.9
High	1	40.2± 0.6	26.6 ± 1.2	29.4±0.4
High	4	41.6± 0.1	27.9± 0.3	16.5±1.6
Medium	0	36.9 ± 0.8	24.5± 0.7	37.9±0.8
Medium	1	37.1± 0.1	21.5± 0.3	31.4±0.6
Medium	4	41.5± 1.7	25.7± 0.3	23.0±0.7

### PGD becomes stiffer during degradation

The polymer is becoming stiffer as it is degrading *in vivo*. Compression testing at 37°C indicates a stiffer polymer after 1 month (p <0.001) and 4 months (p < 0.001) compared to respective non-implanted controls (**Fig 5**). Implant of hPGD are stiffer than mPGD after 1 month of implantation (p<0.01) possibly resulting from a disproportionate degradation rate of amorphous versus crystalline regions. Between 1 and 4 months, hPGD implants become less stiff (p<0.01), indicating further degradation of the crosslinks resulting in more amorphous regions as represented by the decomposition region of the 4 month melt transition curve in **Fig 4A**.

## Discussion

Biodegradable SMEs are an emerging class of biomaterials that demonstrate applicability in various soft tissue repair applications. Although the mechanical properties of PGD at time of implantation may be matched by various formulations of existing biodegradable SMEs, effective biomaterial implant design should consider how the degrading material structure and consequently altered mechanical properties support tissue ingrowth during the entire regenerative timeframe. An ideal material sustains tissue ingrowth and remodeling during the entire healing process thereby matching the degradation rate with the tissue regeneration rate in a given clinical application. The *in vitro* and *in vivo* degradation rate of PGD described in this study, in addition to the changing mechanical properties provide initial insight into the potential clinical performance of implanted PGD based devices for soft tissue regeneration.

### High cure PGD degrades faster *in vitro*

For many elastomeric polymers, *in vitro* degradation rates rarely align with *in* vivo degradation rates primarily due to the differing mechanisms of degradation, differences in fluid flow, and mass transport. Hydrolytic degradation *in vitro* which occurs through a non-biological process can inform polymer design strategies meant to attenuate or facilitate resorption rates by targeting specific modes of biodegradation. Polyester bonds forming the majority of the PGD backbone are subject to hydrolytic degradation. *In vitro* hydrolytic degradation of PGD indicates an initial mass loss of 20% after 2 weeks in PBS, with another 20% after 8 weeks and an additional 30–50% degradation after 18 weeks. Although compounded by enzymatic degradation processes, a similar trend is observed *in vivo* where there is greater than 50% of polymer loss after 8 weeks *in vivo*. The similar trend in degradation rates supports the use of *in vitro* hydrolytic degradation assays to test degradation rates of various designs and formulations of PGD.

### PGD degrades by surface erosion *in vivo*

The concomitant reduction in thickness and diameter, reflected by the proportionate decrease in surface area and volume suggests surface erosion to be the primary mode of *in vivo* biodegradation. Water uptake by hPGD and mPGD scaled with time, but there was a greater swelling after 4 weeks in mPGD compared to hPGD. Similarly, DSC thermograms of the melt transition indicate a prominent crosslinked semicrystalline region followed by an amorphous decomposing region at the 4 month time point further supporting the breakdown of crosslinks. These differences were also observed in other biodegradable elastomers with varying crosslink densities[24]. The increase in water uptake is largely driven by the crosslink density which may decrease disproportionately in less cured materials compared to more cured materials. Consistent with prior studies [7,24], altering PGD curing conditions and thereby changing initial crosslink densities did not significantly affect overall resorption rates of the polymer as indicated by non-significant differences in % mass loss at the 4 month timepoint between mPGD and hPGD. This further suggests the method of degradation to be primarily driven by surface erosion since bulk degradation is largely driven by water infiltration, swelling and softening of the polymer and a breakdown of crosslinks. The increased stiffness of the polymer after 1 month implantation can be attributed to the disproportionate degradation of amorphous versus crystalline and crosslinked regions in the polymer affecting the overall shape recovery transition temperatures. Conventional biodegradable polymers soften as a consequence of hydrolyzing alpha-hydroxy ester bonds. A comparable biodegradable elastomer, poly(glycerol sebacate), also experiences a 50% mass loss within the first 6 weeks, but consequently results in a 20% loss of elastic modulus[23,24]. In contrast, hydrolytic degradation of PGD polymer crosslinks caused an upward shift in the transition temperatures, likely contributing to increased stiffness. An additional explanation of increased stiffness may be attributed to crosslink degradation which increases transition temperatures. PGD is a semi-crystalline branched network held together by a random arrangement of crosslinks. As these crosslinks degrade, regions of the polymer matrix begin to transition at temperatures greater than 37°C indicated by the higher peak T_m_ of both mPGD and hPGD. This increased transition temperature causes certain regions of the polymer to transition back from an elastomeric to a plastic phase resulting in increased stiffness. The variable degradation of amorphous and crystalline regions also can also contribute to the differences in mechanical properties in explanted samples. For instance, more crosslinked hPGD polymer has fewer lamellar regions and more amorphous regions than mPGD at time zero. Softer amorphous regions degrade faster than stiffer crystalline regions resulting in a disproportionate increase in polymer stiffness in hPGD compared to medium cured PGD after one month. There is more swelling in mPGD compared to hPGD between implants after 4 months. Although more water is present in the mPGD polymer, crystallinity of hPGD decreased by a greater extent after 4 months. This greater reduction in crystalline regions of hPGD corresponds with reduced stiffness.

## Conclusion

Biodegradable SMEs are an emerging class of biomaterials for soft tissue repair in large part driven by the suitable mechanical properties of the material in relation to tissue pathology. Characterizing changes in material structural and mechanical properties during the degradation timeframe is of critical importance for a biodegradable material that supports and promotes tissue regeneration. Handling and ease of use in addition to appropriate delivery of the materials is critical for any transplantable materials technologies for regenerative medicine applications. PGD undergoes changes in molecular architecture leading to an increase in transition temperature and degradation consequently increasing the stiffness of the polymer. These findings further emphasize the importance of evaluating changes in transition temperatures of degrading SMEs because dramatic changes in mechanical properties resulting from altered transition temperatures can have catastrophic consequences on soft tissue implants. There will be a growing demand for minimally invasive procedures in large part driven by lower operational costs, shorter length of stay, less adverse events and consequently lower reimbursement costs. Biodegradable SMEs with controlled degradation rates can provide significant handling advantages with specific applications in a variety of minimally invasive soft tissue repair procedures.
